# Deriving analytical solutions using symbolic matrix structural analysis for continuous beams

**DOI:** 10.1038/s41598-025-98023-x

**Published:** 2025-05-07

**Authors:** Vagelis Plevris, Afaq Ahmad

**Affiliations:** 1 Department of Civil and Environmental Engineering, College of Engineering, Qatar University, P.O. Box: 2713, Doha, Qatar; 2 Department of Built Environment, Oslo Metropolitan University, St. Olavs Plass, P.O. Box 4, Oslo, NO-0130 Norway

**Keywords:** Matrix structural analysis, Symbolic, Continuous beams, Structural analysis, Sensitivity analysis, Influence lines, Analytical solutions, Closed-form solutions, Computational science, Civil engineering, Mechanical engineering

## Abstract

This study investigates the use of symbolic computation in Matrix Structural Analysis (MatSA) for continuous beams, using the MATLAB Symbolic Math Toolbox. By employing symbolic MatSA, analytical expressions for displacements, support reactions, and internal forces are derived, offering deeper insights into structural behavior. This approach facilitates efficient and scalable sensitivity analysis, where partial derivatives of outputs concerning input parameters can be directly computed, enhancing design exploration. The development includes an open-source MATLAB program, hosted on GitHub, enabling symbolic analysis of continuous beams subjected to point and uniform loads. This approach is valuable for both engineering practice and pedagogy, enriching the understanding of structural mechanics and aiding in education by illustrating clear parameter relationships. The program supports deriving influence lines and identifying maximum response values.

## Introduction to symbolic matrix structural analysis for structural engineering

The Finite Element Method (FEM), which recently marked its 80th anniversary since its inception Liu, Li^1^, has been a foundational tool in structural analysis, offering a robust numerical framework for solving complex engineering problems^2^. Traditional FEM approaches are predominantly numerical, relying on computations to approximate structural behavior under specified loads and boundary conditions. While effective for producing detailed, problem-specific results, these methods have limitations, particularly in flexibility and generality. Numerical FEM solutions are generally applicable to a specific set of inputs, such as material properties, geometry, and external loads^3^. Modifications to these inputs necessitate a complete re-computation of the system, which can be both time-consuming and computationally intensive, especially for large-scale structures. Additionally, numerical results often obscure the relationships between key parameters, making it difficult to develop a deeper understanding of structural behavior.

FEM applied to linear-type structures, such as beams, trusses, and frames, is commonly referred to as Matrix Structural Analysis (MatSA). Both methods share the same underlying principles and origins. In MatSA, analytical expressions for the stiffness matrix of each element can often be derived directly, bypassing the need for numerical integration techniques such as Gaussian quadrature. This feature enhances the process by making the derivation simpler and more insightful. One of the primary drawbacks of traditional numerical FEM and MatSA is their reliance on predefined boundary conditions and loading scenarios. For every new condition, the entire model must be regenerated and recalculated, leading to a repetitive and time-consuming workflow. This limitation hinders the method’s adaptability and restricts its application in tasks requiring real-time analysis or optimization^4^, where multiple configurations need to be evaluated quickly and efficiently^5^.

Symbolic computation offers a promising alternative to these challenges. Unlike numerical computation, which yields specific numerical values, symbolic computation allows for the manipulation and solution of mathematical expressions in their exact, algebraic form. This approach opens new avenues for deriving analytical solutions and gaining deeper insights into structural behavior.

MATLAB is extensively utilized in structural engineering due to its robust numerical algorithms, which enable the resolution of a variety of engineering challenges, including matrix analysis of structures^6–8^, structural dynamics^9–11^, structural optimization^12–14^, and more^15^. MATLAB is primarily known for its numerical computing capabilities, but it also includes features for symbolic computation. The MATLAB Symbolic Math Toolbox^16^ significantly enhances MATLAB’s functionality, allowing it to handle symbolic computation^17,18^. The toolbox supports a wide range of symbolic operations, including algebraic simplifications, differentiation, integration, equation solving, and matrix manipulation^19^. It is particularly valuable in engineering, physics, and mathematics, where it facilitates the analysis of complex systems by providing exact, parameterized solutions that can be easily manipulated and interpreted. For researchers and educators, the Symbolic Math Toolbox is a useful resource, as it enhances the ability to explore theoretical concepts, derive closed-form solutions, and present results in a more intuitive and generalizable manner. Its integration with the program’s numerical environment also allows for seamless transitions between symbolic and numerical analysis, offering a comprehensive platform for tackling both theoretical and practical problems.

Other software and programming languages like Mathematica^20^, Maple^21^, and SymPy (Python)^22^ also offer symbolic computation capabilities. This study focuses on the MATLAB Symbolic Math Toolbox^16,23^, which was used to develop the symbolic analysis open-source code of this work. However, similar programs can be developed using these alternative platforms, following the same underlying principles. In structural analysis, the toolbox facilitates the expression of MatSA solutions in symbolic form, where the system’s response is represented by algebraic expressions involving key parameters related to material or section properties. This method is beneficial for analyzing structural elements such as beams, trusses, and frames, where the stiffness matrix for each element can be derived symbolically, eliminating the need for complex numerical integration. By combining the symbolic stiffness matrices of individual elements and expressing the force vector symbolically, fully symbolic solutions for displacements, element internal forces, or support reactions can be obtained. This enables flexible manipulation of solutions to accommodate various loading conditions, boundary constraints, or material properties without the need for repeated recalculations. A preprint of this work can be found in^24^. The second part of this work, which is about the analysis of plane trusses has been published in^25,26^.

### Literature review

There have been relatively few attempts in the literature to address stiffness matrices symbolically. Korncoff and Fenves^27^ made an early effort to develop a symbolic processor aimed at assisting in the generation of stiffness matrices for finite elements, despite the limited computational resources available at the time. Their results, however, highlighted several promising avenues for future research, both within the specific domain of finite element analysis (FEA) and in the broader application of symbolic processing techniques. Leff and Yun^28^ presented a system for generating global stiffness matrices where elements are expressed as functions of shape parameters. Their approach builds on STRUDL syntax, but with a significant difference: instead of fixed values, joint coordinates, material properties, and forces are entered as parameterized expressions. The resulting stiffness matrix retains these parameter dependencies, providing flexibility in the analysis. This method allows engineers to explore how changes in structural parameters affect the stiffness matrix, offering a more versatile and adaptive framework for FEA.

Nagabhushanam, Srinivas^29^ developed a specialized symbolic manipulation package in FORTRAN for generating elemental matrices in FEA. The package allows users to perform symbolic manipulations through simple, user-friendly commands. With a modular structure and minimal memory requirements, it efficiently handles large-order matrices, even on personal computers. The package supports various element geometries and shape functions, including isoparametric elements, and offers a multilevel operator facility to perform several manipulations with a single command. This compact tool has been successfully applied to generate elemental stiffness, flexibility, and nodal force matrices, demonstrating its utility in symbolic FEA. Tummarakota and Lieh^30^ addressed the need for efficient algorithms to model and predict the behavior of multibody structural systems in applications such as aerospace, robotics, and automotive engineering. They utilized a computer-aided symbolic method to formulate equations of motion based on Lagrange’s method, offering greater physical insight compared to traditional numerical approaches. The generated equations are automatically converted into FORTRAN code, enabling simulations and control synthesis. The study demonstrated the effectiveness of this approach through two examples: a slider-crank mechanism and an aircraft model, which are solved using the Runge-Kutta-Fehlberg method for numerical integration.

Eriksson and Pacoste^31^ discuss the use of symbolic software in developing finite element procedures, particularly for complex problems involving higher-order instabilities requiring precise formulations. The authors highlight that symbolic tools enhance the efficiency and clarity of procedural development, enabling effective comparisons between various element assumptions. The research includes beam formulations for plane and space models, allowing analytical verification of equivalence between displacement and co-rotational contexts. Symbolic derivation also simplifies handling finite space rotations and supports the systematic derivation of local displacements from global variables. Amberg, Tönhardt^32^ describe the development of a toolbox in Maple for generating finite element codes from symbolic mathematical specifications, facilitating 1D, 2D, and 3D simulations. This toolbox has significantly accelerated research in areas such as thermocapillary convection, welding, and crystal growth by reducing the time from conceptualization to a functioning simulation to mere hours. The approach promotes flexibility, transparency, and thorough documentation, enabling researchers to easily modify models and focus on physical insights and numerical properties while minimizing errors and debugging.

Pavlović^33^ discussed the use of symbolic computation in structural engineering, highlighting its emergence as a powerful tool alongside traditional numerical methods. He reviewed past applications where symbolic computations have been underutilized and emphasized the potential for significant advancements in areas of classical structural analysis. The author argues that symbolic computation invigorates classical analytical techniques, offering new opportunities to address complex structural problems. He proposes a more balanced approach that integrates both symbolic and numerical methods, demonstrating their complementary strengths in solving structural mechanics problems efficiently and accurately.

Skrinar and Pliberšek^34^ derived a symbolic stiffness matrix and load vector for a slender beam with multiple transverse cracks under uniform loading. Using the principle of virtual work, they provided closed-form analytical expressions that facilitate fast and straightforward evaluations. This approach, which excludes shape functions, makes the impact of crack depth and location on flexural deformation clearer, aiding in crack identification. The developed matrix is ideal for modeling flexural cracks in beams and columns, which is relevant in earthquake engineering per European design code EC8. Roque^35^ explored the symbolic and numerical analysis of bending plates using MATLAB for symbolic manipulation of expressions. The author emphasized the importance of integrating numerical and symbolic approaches in problem-solving, highlighting MATLAB’s versatility in seamlessly combining these computational methods.

### Symbolic vs. Numerical expressions and solutions in MatSA

The symbolic representation of structural analysis problems can provide engineers and researchers with deeper insight into structural behavior. By maintaining algebraic relationships between parameters, symbolic MatSA enables the exploration of how changes in material properties, geometry, or loading affect the overall response of the structure. Symbolic solutions are particularly useful in scenarios where flexibility and adaptability are crucial, as they provide parameterized solutions not confined to specific input values. Furthermore, when symbolic expressions are available, engineers can easily calculate partial derivatives with respect to various input parameters, facilitating sensitivity analyses and design optimization in a straightforward and efficient manner, as will be examined in detail in Sect. 6.

One of the key advantages of symbolic MatSA lies in its educational value. Symbolic solutions can help students and professionals develop a deeper understanding of the fundamental principles of mechanics and structural behavior. By maintaining algebraic relationships between key parameters, such as the modulus of elasticity or the moment of inertia of the beam’s cross-section, symbolic expressions explicitly reveal how these factors influence displacements, stresses, and strains. This level of transparency enhances conceptual learning and can provide insights that may be less apparent in purely numerical approaches, which often produce results without explicitly showing the underlying parameter dependencies. However, it is important to recognize that numerical methods remain essential in structural analysis, particularly for solving large-scale problems with thousands or even millions of unknowns, which are beyond the reach of any symbolic computation. In this regard, symbolic and numerical approaches should be seen as complementary rather than competing techniques, each offering unique advantages depending on the context and scale of the problem at hand.

### Key features and novelty of this study

This study introduces an open-source MATLAB program designed for symbolic MatSA of continuous beams under point and uniform loads. While previous research has explored symbolic solutions for beams, to the best of our knowledge, this is the first program capable of automatically deriving analytical solutions for any continuous beam configuration, regardless of its complexity. Users can define beam problems to obtain symbolic expressions for displacements, forces, and moments at any position *x* or determine their maximum values efficiently. The main features of the program include:

- Closed-form solutions for any output quantities (e.g., support reactions) across various continuous beam configurations.
- Solutions expressed as functions of *x* for specific points on the beam (e.g., bending moment *M*(*x*)) and determination of maximum values along the beam (e.g., *M*_max_).
- Analytical expressions for influence lines for specified outputs (e.g., support reactions influenced by a unit load moving along the beam).
- Sensitivity analysis for any output quantity relative to input parameters, utilizing MATLAB’s built-in differentiation capabilities.

The source code is freely available at GitHub (https://github.com/vplevris/SymbolicMatSA-Beams), accompanied by all examples discussed in the study. This work was implemented using MATLAB R2024b and its symbolic toolbox, and it is anticipated to be compatible with earlier versions. Users are encouraged to download the code, experiment, test it, and create their own analytical solutions. The implementation is user-friendly, requiring minimal setup to execute custom analyses.

## Continuous beam stiffness matrix

**Fig. 1:**
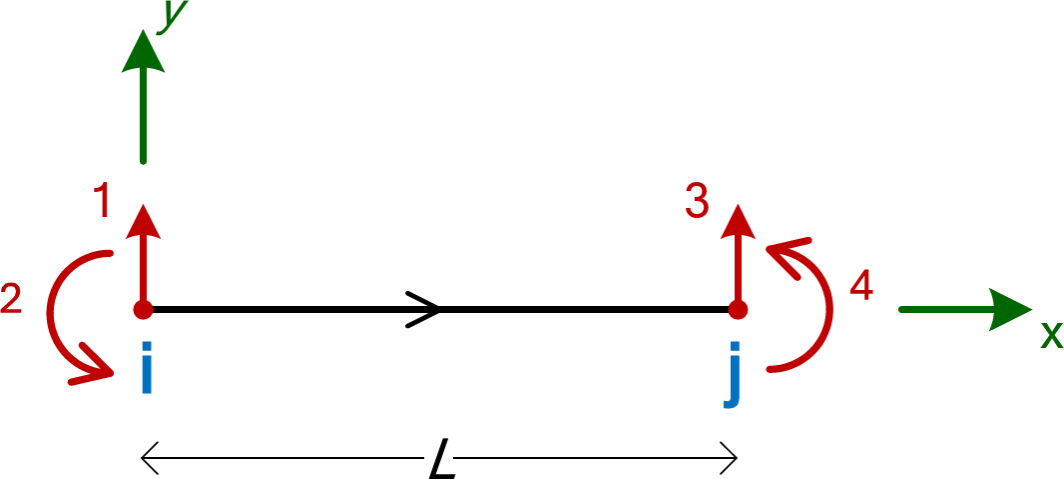
2D Euler–Bernoulli beam element with 4 DOFs.

The stiffness matrix plays a crucial role in MatSA as it defines the relationship between the applied forces and the resulting displacements within a structural system. Typically, in MatSA/FEM stiffness matrices are computed numerically for each individual element and subsequently assembled into a global system of equations representing the entire structure. Numerical integration is usually needed, for example for the case of 2D plane stress elements or more complex elements, which is regularly done using Gauss quadrature. However, in MatSA, for linear-type elements, it is usually possible to derive an exact symbolic expression for the stiffness matrix of an element. One such case is the 2D Euler–Bernoulli beam with 3 degrees of freedom (DOFs) per node^15^ which is commonly used in the linear static analysis of plane beams and frames. In this study, we use a beam element with 2 DOFs per node, one translational and one rotational, resulting in 4 DOFs for the element, as shown in Fig. 1. The axial degree of freedom is omitted because the specific element is primarily designed for the analysis of continuous beams, where axial effects are typically negligible.

In this context, calculating the element stiffness matrix is straightforward. The symbolic 4 × 4 stiffness matrix for the Euler-Bernoulli beam element, assuming the axial degree of freedom is neglected, corresponding to the DOFs shown in Fig. 1, is given by Eq. (1).

1 $$[\hat{k}_{{b2}} ] = \frac{{EI}}{{L^{3} }} \cdot \left[ {\begin{array}{*{20}c} {12} & {6L} & { - 12} & {6L} \\ {6L} & {4L^{2} } & { - 6L} & {2L^{2} } \\ { - 12} & { - 6L} & {12} & { - 6L} \\ {6L} & {2L^{2} } & { - 6L} & {4L^{2} } \\ \end{array} } \right]$$

In this expression, *E* denotes Young’s modulus of the material, *I* represents the moment of inertia of the beam’s cross-section, and *L* is the length of the beam element. For continuous beam analysis, where all beam elements are essentially horizontal and oriented from the left (Start node i) to the right (End node j), the local stiffness matrix coincides with the global stiffness matrix of the element. This eliminates the need for any element transformation, as the rotation angle of the element is zero.

## Educational benefits and importance of symbolic solutions in structural analysis

Symbolic solutions in MatSA provide significant educational advantages by maintaining explicit relationships between structural parameters. Unlike numerical methods, which yield specific values for given inputs, symbolic solutions express results in algebraic form, making it easier to understand how variables such as modulus of elasticity (*E*), moment of inertia (*I*), and span length (*L*) influence displacements, forces, and moments. This clarity enhances both theoretical understanding and practical applications in structural analysis.

A key benefit of symbolic MatSA is its ability to visualize parameter dependencies. In traditional numerical solutions, results are obtained for specific cases, only. Symbolic expressions explicitly reveal how different parameters contribute to structural behavior. For instance, in a simply supported beam with a point load at midspan, the displacement formula is

2 $$U=\frac{{P{L^3}}}{{48EI}}$$

From this, students and engineers can immediately see that increasing the span length or reducing material stiffness significantly increases displacement. Such direct relationships are not readily apparent in numerical approaches, where multiple simulations with varying input values are required to observe trends.

Another important aspect of symbolic solutions is their role in superposition and structural behavior analysis. When analyzing continuous beams subjected to multiple loads, symbolic expressions make it possible to isolate the effect of each load independently and then combine them. This is particularly useful in understanding load interaction effects and verifying results in structural mechanics courses.

Another advantage of symbolic MatSA is its usefulness in structural design education. Many classical structural engineering problems involve parameterized solutions that help students grasp the effects of geometric, material, and loading variations. Instructors can use symbolic solutions to illustrate fundamental principles of mechanics, allowing students to explore how different design choices impact structural response. The ability to manipulate symbolic expressions brings deeper engagement with structural mechanics, reinforcing key concepts through direct algebraic relationships rather than isolated numerical outputs. The use of symbolic computation in structural analysis education aligns with findings from other educational research. For instance, the novel Image-based Structural Analysis method demonstrates how integrating technology can enhance the learning experience by allowing students to analyze structural responses directly from hand-drawn sketches^36^.

Although symbolic analysis offers clarity and insight, numerical methods remain essential for large-scale structural problems, where symbolic computation is impractical due to the number of unknowns. Numerical FEA can efficiently handle complex structures with thousands of degrees of freedom, making it the preferred choice for real-world full-scale engineering applications. However, symbolic and numerical methods are not competing but complementary approaches—symbolic solutions provide deeper understanding and theoretical insight, while numerical methods enable large-scale computations and real-world applications.

In summary, symbolic MatSA bridges the gap between theory and computation by providing exact, parameterized solutions that enhance structural analysis education. The ability to explicitly express interdependencies between input parameters and structural responses makes symbolic methods a powerful tool for both learning and research. When used alongside numerical methods, they enable a comprehensive approach to structural analysis, equipping engineers and students with the tools needed for both theoretical exploration and practical application.

## Symbolic expressions for intermediate values of internal forces and local extrema

In matrix structural analysis of beams, whether conducted numerically or symbolically, the results typically include displacements (or rotations) at nodal points and element forces at the ends of each element (nodes i and j). Although intermediate values for displacements or internal forces are not provided directly by the method, these quantities can be determined indirectly by applying established principles of statics.

**Fig. 2:**
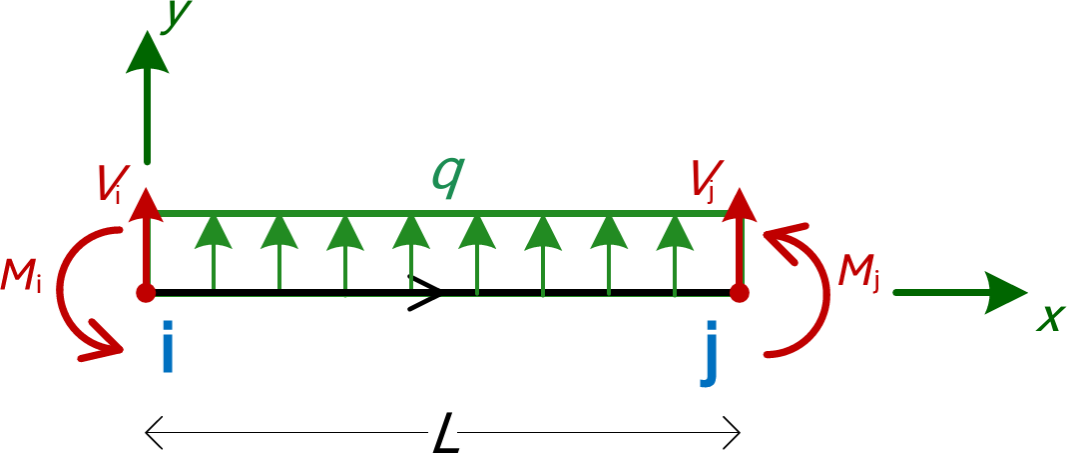
2D beam element with uniform load *q* on.

It is quite straightforward to find the intermediate values of internal forces (shear force and bending moment) at any given point *x* within an element, based on equilibrium and the known forces at the ends. We consider the general case of a beam element with uniform load *q* on it (positive *q* points upwards, towards the *y* axis of the beam), as shown in Fig. 2.

The formulas for the internal shear force and the bending moment at a given location, *x*, calculated from the left side, are:

3 $$V(x)={V_i}+q \cdot x$$

4 $$M(x)= - \left( {{M_i} - {V_i} \cdot x - q\frac{{{x^2}}}{2}} \right)=q\frac{{{x^2}}}{2}+{V_i} \cdot x - {M_i}$$

**Fig. 3:**
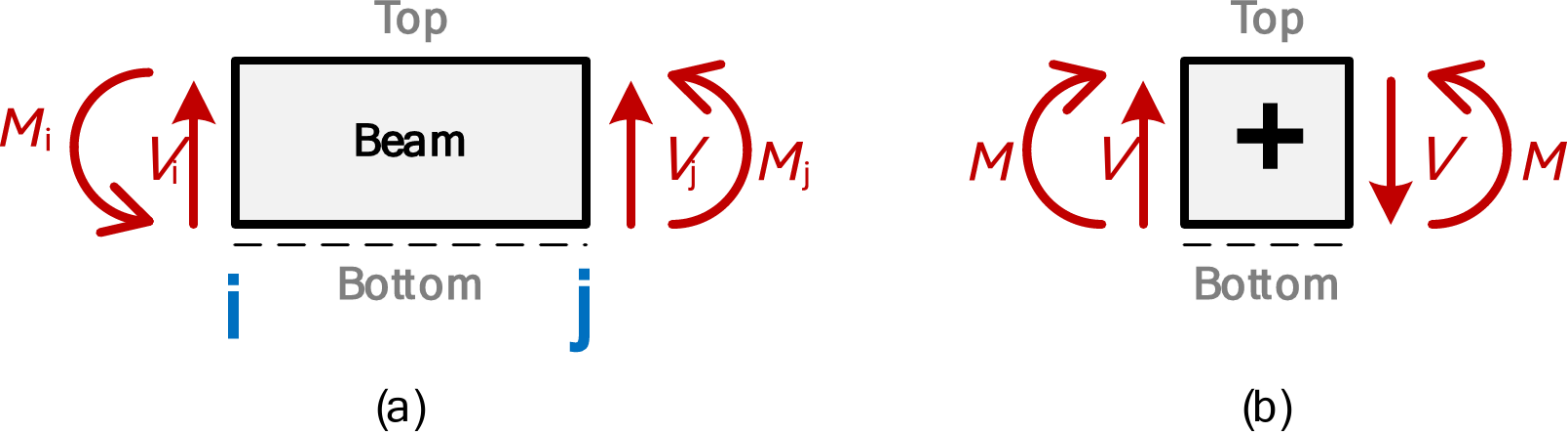
Positive notation used (**a**) For the MatSA results at each end of the beam (i and j), (**b**) For the shear force and bending moment diagrams.

Here, *x* represents the distance along the local element *x*-axis, measured from the left node (Node i) of the element. When *x* = 0, it corresponds to the starting point of the element (Node i), while *x* = *L* marks the end of the element (Node j). The end moments, *M*_i_ and *M*_j_, are defined as positive when they act counter-clockwise, consistent with the notation in Fig. 1. On the other hand, for the shear force and bending moment diagrams, the internal shear force *V*(*x*) is positive when it induces clockwise rotation of the element, and the internal bending moment *M*(*x*) is considered positive when it induces tension in the bottom fiber of the beam. These conventions are detailed in Fig. 3(a) and Fig. 3(b). In Eq. (4), the internal bending moment is calculated from the left side, necessitating a negative sign to align with this convention and ensure positivity when it induces tension in the bottom fiber, as illustrated in Fig. 3(b).

For the case of a uniform load (i.e. *q* ≠ 0), the extremum (maximum or minimum) bending moment is typically found at the point where the shear force equals zero, i.e., at

5 $${x_{extrM}}= - \frac{{{V_i}}}{q}$$

And according to Eq. (4) the value of the extremum bending moment will then be

6 $$M({x_{extrM}})= - {M_i}+{V_i} \cdot \left( { - \frac{{{V_i}}}{q}} \right)+q\frac{{{{\left( { - \frac{{{V_i}}}{q}} \right)}^2}}}{2}= - {M_i} - \frac{{V_{i}^{2}}}{{2q}}$$

## Numerical examples

We consider five numerical examples of differing levels of complexity. In these, *EI* is treated as a single symbolic parameter, since *E* and *I* consistently appear together in stiffness and other analytical expressions. The first three examples have only point loads, while the other two have uniform loads. Tables 1 and 2 provide detailed descriptions of the five examples and the associated symbolic parameters for each.

**Table 1:** Details of the numerical examples 1, 2, and 3.

Example #	Figure	Symbolic Parameters
1		3 (*EI*, *L*, and *P*)
2		4 (*EI*, *L*, *a*, and *P*)
3		5 (*EI*, *a*, *b*, *P*_1_, and *P*_2_)

**Table 2:** Details of the numerical examples 4 and 5.

Example #	Figure	Symbolic Parameters
4		5 (*EI*, *L*, *a*, *b*, and *w*)
5		3 (*EI*, *L*, and *w*)

### Simply supported beam with a point load (3 symbolic Parameters)

**Fig. 4:**
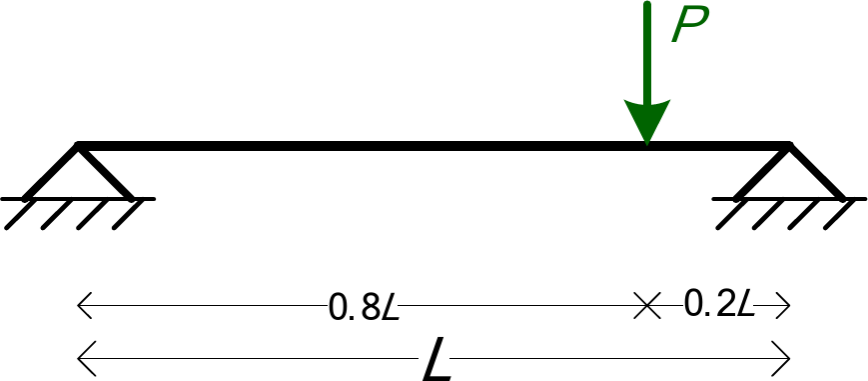
The beam of Example 1.

The first numerical example is a simply supported beam with a point load *P* applied at *x* = 0.8*L*, as shown in Fig. 4. The symbolic parameters are three: *EI*, *L*, and *P*.

Table 3 shows the details of the model, as given in MATLAB. In every example, the number of elements (*NumElements*) is defined by the size of the *Lengths* vector and the number of nodes (*NumNodes*) is (*NumElements*-1). In this example we have 2 elements and 3 nodes. Note that a node is needed to define any point load.

**Table 3:** Details of the input parameters of the 1st numerical example.

Lengths	$${\left[ {\begin{array}{*{20}{c}} {0.8 \cdot L}&{0.2 \cdot L} \end{array}} \right]^T}$$
Supports	$${\left[ {\begin{array}{*{20}{c}} 1&0&1 \\ 0&0&0 \end{array}} \right]^T}$$
PointLoads	$${\left[ {\begin{array}{*{20}{c}} 0&{ - P}&0 \\ 0&0&0 \end{array}} \right]^T}$$
UniformLoads	$${\left[ {\begin{array}{*{20}{c}} 0&0 \end{array}} \right]^T}$$

Tables 4, 5 and 6 present the results of the symbolic analysis in terms of the symbolic parameters. Table 4 presents the Node displacements and rotations, while Table 5 shows the support reactions and Table 6 the element forces and moments at the start (i) and end (j) of each element.

**Table 4:** Example 1: node displacements and rotations.

Node #	y-Displacement (D_y_)	z-Rotation (*R*_z_)
Node 1	0	$- \frac{{4P{L^2}}}{{125EI}}$
Node 2	$- \frac{{16P{L^3}}}{{1875EI}}$	$\frac{{4P{L^2}}}{{125EI}}$
Node 3	0	$\frac{{6P{L^2}}}{{125EI}}$

**Table 5:** Example 1: support reactions.

Node #	Force F_y_	Moment M_z_
Node 1	${P \mathord{\left/ {\vphantom {P 5}} \right. \kern-0pt} 5}$	-
Node 3	${{4P} \mathord{\left/ {\vphantom {{4P} 5}} \right. \kern-0pt} 5}$	-

**Table 6:** Example 1: element forces and bending moments.

Element #	Start / End	Shear Force (V_i_, V_j_)	Moment (M_i_, M_j_)
1	Start (i)	${P \mathord{\left/ {\vphantom {P 5}} \right. \kern-0pt} 5}$	0
	End (j)	${{ - P} \mathord{\left/ {\vphantom {{ - P} 5}} \right. \kern-0pt} 5}$	${{4PL} \mathord{\left/ {\vphantom {{4PL} {25}}} \right. \kern-0pt} {25}}$
2	Start (i)	${{ - 4P} \mathord{\left/ {\vphantom {{ - 4P} 5}} \right. \kern-0pt} 5}$	${{ - 4PL} \mathord{\left/ {\vphantom {{ - 4PL} {25}}} \right. \kern-0pt} {25}}$
	End (j)	${{4P} \mathord{\left/ {\vphantom {{4P} 5}} \right. \kern-0pt} 5}$	0

### Fixed-End beam with point load (4 symbolic Parameters)

**Fig. 5:**
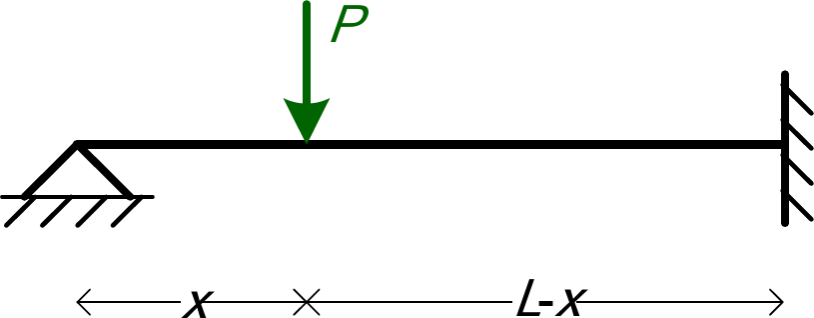
The beam of Example 2.

The second numerical example is a fixed-end beam with a point load *P*, as shown in Fig. 5. The symbolic parameters are four: *EI*, *L*, *x*, and *P*. This example will also serve to illustrate the concept of influence lines.

Table 7 shows the details of the model, as given in MATLAB. In this example we have 3 nodes and 2 elements.

**Table 7:** Details of the input parameters of the 2nd numerical example.

Lengths	$${\left[ {\begin{array}{*{20}{c}} x&{L - x} \end{array}} \right]^T}$$
Supports	$${\left[ {\begin{array}{*{20}{c}} 1&0&1 \\ 0&0&1 \end{array}} \right]^T}$$
PointLoads	$${\left[ {\begin{array}{*{20}{c}} 0&{ - P}&0 \\ 0&0&0 \end{array}} \right]^T}$$
UniformLoads	$${\left[ {\begin{array}{*{20}{c}} 0&0 \end{array}} \right]^T}$$

The results of the symbolic analysis are given in Tables 8 and 9, and Table 10, for Node displacements and rotations; support reactions; and element forces and moments, respectively.

**Table 8:** Example 2: node displacements and rotations.

Node #	y-Displacement (D_y_)	z-Rotation (*R*_z_)
Node 1	0	$- \frac{{Px{{(L - x)}^2}}}{{4EIL}}$
Node 2	$- \frac{{Px^{2} (L - x)^{3} (3L + x)}}{{12EIL^{3} }}$	$\frac{{Px{{(L - x)}^2}( - {L^2}+2Lx+{x^2})}}{{4EI{L^3}}}$
Node 3	0	0

**Table 9:** Example 2: support reactions.

Node #	Force F_y_	Moment M_z_
Node 1	$\frac{{P(L - x)^{2} (2L + x)}}{{2L^{3} }}$	-
Node 3	$\frac{{Px(3{L^2} - {x^2})}}{{2{L^3}}}$	$- \frac{{Px({L^2} - {x^2})}}{{2{L^2}}}$

**Table 10:** Example 2: element forces and bending moments.

Element #	Start / End	Shear Force (V_i_, V_j_)	Moment (M_i_, M_j_)
1	Start (i)	$\frac{{P(L - x)^{2} (2L + x)}}{{2L^{3} }}$	0
	End (j)	$- \frac{{P(L - x)^{2} (2L + x)}}{{2L^{3} }}$	$\frac{{Px(L - x)^{2} (2L + x)}}{{2L^{3} }}$
2	Start (i)	$- \frac{{Px(3{L^2} - {x^2})}}{{2{L^3}}}$	$- \frac{{Px(L - x)^{2} (2L + x)}}{{2L^{3} }}$
	End (j)	$\frac{{Px(3{L^2} - {x^2})}}{{2{L^3}}}$	$- \frac{{Px({L^2} - {x^2})}}{{2{L^2}}}$

Now suppose we would like to draw the influence line of the reaction moment at Node 3. According to the results shown in Table 9, the reaction moment at Node 3 (counter-clockwise is positive) is given by

7 $${M_{z,3}}= - \frac{{Px({L^2} - {x^2})}}{{2{L^2}}}$$

To draw the influence line, we assume a unit load (*P* = 1) applied along the beam. For illustration purposes, we will assume that the length of the beam is also unitary (*L* = 1). Then the reaction moment is given by the formula:

8 $${M_{z,3}}= - \frac{{x(1 - {x^2})}}{2}$$

**Fig. 6:**
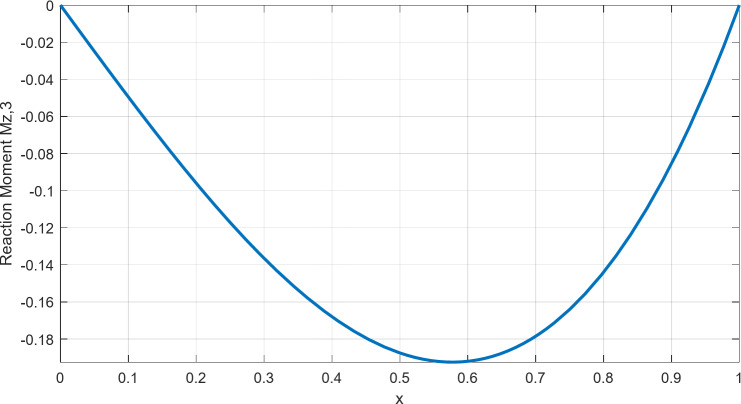
The influence line of the reaction moment at Node 3, for Example 3.

The influence line corresponding to Eq. (8) is presented in Fig. 6.

We observe that the reaction moment takes the value of zero when the load is at the beginning (*x* = 0) or the end (*x* = *L* = 1) of the beam, while it takes its maximum (in absolute terms) value ${M_{\hbox{max} }}={{ - \sqrt 3 } \mathord{\left/ {\vphantom {{ - \sqrt 3 } 9}} \right. \kern-0pt} 9} \approx - 0.1925$ when the unitary load is at $x={{\sqrt 3 } \mathord{\left/ {\vphantom {{\sqrt 3 } 3}} \right. \kern-0pt} 3} \approx 0.5774$. Influence lines are essential in bridge design and structural assessments, helping engineers identify critical loading positions for maximum forces and moments. They are widely used in bridge load rating to determine worst-case traffic effects and in structural monitoring to detect potential weaknesses by comparing theoretical and measured responses.

### Two-Span beam with two point loads (5 symbolic Parameters)

**Fig. 7:**
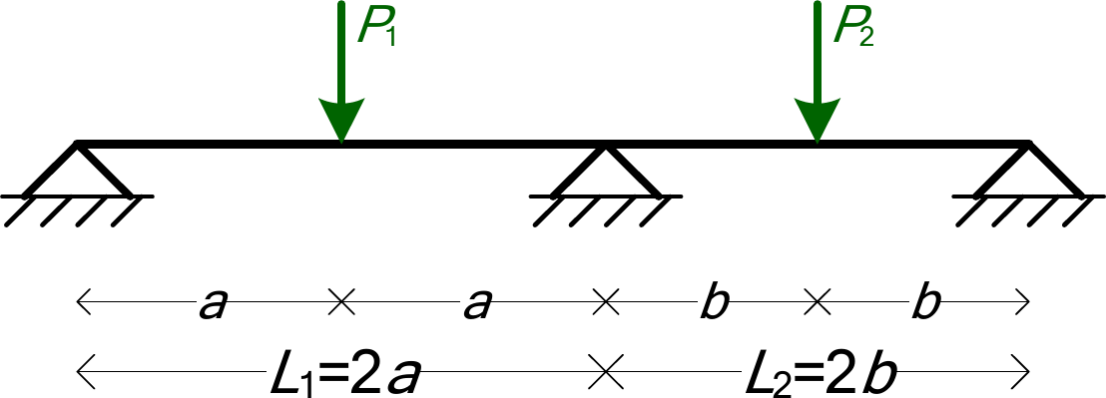
The two-span beam of Example 3.

The third numerical example is a two-span beam with two point loads, as shown in Fig. 7. The symbolic parameters are six: *EI*, *a*, *b*, *P*_1_, and *P*_2_.

Table 11 shows the details of the model, as given in MATLAB.

**Table 11:** Details of the input parameters of the 3rd numerical example.

Lengths	$${\left[ {\begin{array}{*{20}{c}} a&a&b&b \end{array}} \right]^T}$$
Supports	$${\left[ {\begin{array}{*{20}{c}} 1&0&1&0&1 \\ 0&0&0&0&0 \end{array}} \right]^T}$$
PointLoads	$${\left[ {\begin{array}{*{20}{c}} 0&{ - {P_1}}&0&{ - {P_2}}&0 \\ 0&0&0&0&0 \end{array}} \right]^T}$$
UniformLoads	$${\left[ {\begin{array}{*{20}{c}} 0&0&0&0 \end{array}} \right]^T}$$

The results of the symbolic analysis are reported in Tables 12, 13 and 14, for Node displacements and rotations; support reactions; and element forces and moments, respectively.

**Table 12:** Example 3: node displacements and rotations.

Node #	y-Displacement (D_y_)	z-Rotation (*R*_z_)
Node 1	0	$- \frac{{a({P_1}{a^2}+2{P_1}ab - {P_2}{b^2})}}{{8EI(a+b)}}$
Node 2	$- \frac{{{a^2}(7{P_1}{a^2}+16{P_1}ab - 9{P_2}{b^2})}}{{96EI(a+b)}}$	$\frac{{a({P_1}{a^2}+{P_2}{b^2})}}{{32EI(a+b)}}$
Node 3	0	$\frac{{ab\left( {{P_1}a - {P_2}b} \right)}}{{4EI(a+b)}}$
Node 4	$- \frac{{{b^2}( - 9{P_1}{a^2}+16{P_2}ab+7{P_2}{b^2})}}{{96EI(a+b)}}$	$- \frac{{b({P_1}{a^2}+{P_2}{b^2})}}{{32EI(a+b)}}$
Node 5	0	$\frac{{b( - {P_1}{a^2}+2{P_2}ab+{P_2}{b^2})}}{{8EI(a+b)}}$

**Table 13:** Example 3: support reactions.

Node #	Force F_y_	Moment M_z_
Node 1	$\frac{{5{P_1}{a^2}+8{P_1}ab - 3{P_2}{b^2}}}{{16a(a+b)}}$	-
Node 3	$\frac{{3{P_1}{a^2}+3{P_2}{b^2}+8{P_1}ab+8{P_2}ab}}{{16ab}}$	-
Node 5	$\frac{{ - 3{P_1}{a^2}+8{P_2}ab+5{P_2}{b^2}}}{{16b(a+b)}}$	-

**Table 14:** Example 3: element forces and bending moments.

Element #	Start / End	Shear Force (V_i_, V_j_)	Moment (M_i_, M_j_)
1	Start (i)	$\frac{{5{P_1}{a^2}+8{P_1}ab - 3{P_2}{b^2}}}{{16a(a+b)}}$	0
	End (j)	$- \frac{{5{P_1}{a^2}+8{P_1}ab - 3{P_2}{b^2}}}{{16a(a+b)}}$	$\frac{{5{P_1}{a^2}+8{P_1}ab - 3{P_2}{b^2}}}{{16(a+b)}}$
2	Start (i)	$- \frac{{11{P_1}{a^2}+8{P_1}ab+3{P_2}{b^2}}}{{16a(a+b)}}$	$- \frac{{5{P_1}{a^2}+8{P_1}ab - 3{P_2}{b^2}}}{{16(a+b)}}$
	End (j)	$\frac{{11{P_1}{a^2}+8{P_1}ab+3{P_2}{b^2}}}{{16a(a+b)}}$	$- \frac{{3({P_1}{a^2}+{P_2}{b^2})}}{{8(a+b)}}$
3	Start (i)	$\frac{{3{P_1}{a^2}+8{P_2}ab+11{P_2}{b^2}}}{{16b(a+b)}}$	$\frac{{3({P_1}{a^2}+{P_2}{b^2})}}{{8(a+b)}}$
	End (j)	$- \frac{{3{P_1}{a^2}+8{P_2}ab+11{P_2}{b^2}}}{{16b(a+b)}}$	$\frac{{ - 3{P_1}{a^2}+8{P_2}ab+5{P_2}{b^2}}}{{16(a+b)}}$
4	Start (i)	$- \frac{{ - 3{P_1}{a^2}+8{P_2}ab+5{P_2}{b^2}}}{{16b(a+b)}}$	$- \frac{{ - 3{P_1}{a^2}+8{P_2}ab+5{P_2}{b^2}}}{{16(a+b)}}$
	End (j)	$\frac{{ - 3{P_1}{a^2}+8{P_2}ab+5{P_2}{b^2}}}{{16b(a+b)}}$	0

### Simply supported beam with uniform load (5 symbolic Parameters)

**Fig. 8:**
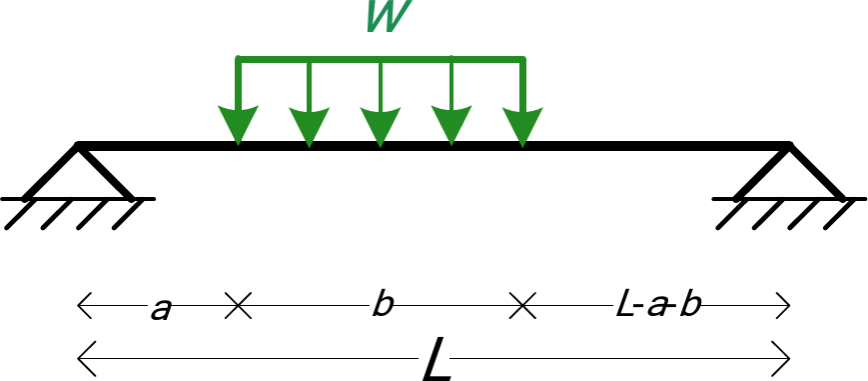
The beam of Example 4.

The fourth numerical example is a simply supported beam with a uniform load *w*, as shown in Fig. 8. The symbolic parameters are five: *EI*, *L*, *a*, *b*, and *w*.

Table 15 shows the details of the model, as given in MATLAB. In this example we have 4 nodes and 3 elements.

**Table 15:** Details of the input parameters of the 4th numerical example.

Lengths	$${\left[ {\begin{array}{*{20}{c}} a&b&{L - a - b} \end{array}} \right]^T}$$
Supports	$${\left[ {\begin{array}{*{20}{c}} 1&0&0&1 \\ 0&0&0&0 \end{array}} \right]^T}$$
PointLoads	$${\left[ {\begin{array}{*{20}{c}} 0&0&0&0 \\ 0&0&0&0 \end{array}} \right]^T}$$
UniformLoads	$${\left[ {\begin{array}{*{20}{c}} 0&{ - w}&0 \end{array}} \right]^T}$$

The results of the symbolic analysis are given in Tables 16 and 17, and Table 18, for Node displacements and rotations; support reactions; and element forces and moments, respectively.

**Table 16:** Example 4: node displacements and rotations.

Node #	y-Displacement (D_y_)	z-Rotation (*R*_z_)
Node 1	0	$- \frac{{bw(8L^{2} a + 4L^{2} b - 12La^{2} - 12Lab - 4Lb^{2} + 4a^{3} + 6a^{2} b + 4ab^{2} + b^{3} )}}{{24EIL}}$
Node 2	$- \frac{{abw(8L^{2} a + 4L^{2} b - 16La^{2} - 12Lab - 4Lb^{2} + 8a^{3} + 8a^{2} b + 4ab^{2} + b^{3} )}}{{24EIL}}$	$- \frac{{bw(8L^{2} a + 4L^{2} b - 24La^{2} - 12Lab - 4Lb^{2} + 16a^{3} + 12a^{2} b + 4ab^{2} + b^{3} )}}{{24EIL}}$
Node 3	$- \frac{{bw(2a+b)(a - L+b)(4{a^2}+6ab - 4La+3{b^2} - 4Lb)}}{{24EIL}}$	$- \frac{{bw(2a+b)(4{L^2} - 12La - 12Lb+8{a^2}+14ab+7{b^2})}}{{24EIL}}$
Node 4	0	$- \frac{{bw(2a+b)( - 2{L^2}+2{a^2}+2ab+{b^2})}}{{24EIL}}$

**Table 17:** Example 4: support reactions.

Node #	Force F_y_	Moment M_z_
Node 1	$- \frac{{bw\left( {2a{\text{ }} - {\text{ }}2L{\text{ }} + {\text{ }}b} \right)}}{{2L}}$	-
Node 4	$\frac{{bw\left( {2a{\text{ }} + {\text{ }}b} \right)}}{{2L}}$	-

**Table 18:** Example 4: element forces and bending moments.

Element #	Start / End	Shear Force (V_i_, V_j_)	Moment (M_i_, M_j_)
1	Start (i)	$- \frac{{bw(2a{\text{ }} - {\text{ }}2L{\text{ }} + {\text{ }}b)}}{{2L}}$	0
	End (j)	$\frac{{bw(2a{\text{ }} - {\text{ }}2L{\text{ }} + {\text{ }}b)}}{{2L}}$	$- \frac{{abw(2a - 2L + b)}}{{2L}}$
2	Start (i)	$- \frac{{bw(2a{\text{ }} - {\text{ }}2L{\text{ }} + {\text{ }}b)}}{{2L}}$	$\frac{{abw(2a - 2L + b)}}{{2L}}$
	End (j)	$\frac{{bw(2a{\text{ }} + {\text{ }}b)}}{{2L}}$	$- \frac{{bw\left( {2a + b} \right)\left( {a - L + b} \right)}}{{2L}}$
3	Start (i)	$- \frac{{bw(2a{\text{ }} + {\text{ }}b)}}{{2L}}$	$\frac{{bw\left( {2a + b} \right)\left( {a - L + b} \right)}}{{2L}}$
	End (j)	$\frac{{bw(2a{\text{ }} + {\text{ }}b)}}{{2L}}$	0

The first three numerical examples did not include any uniform loads. In this fourth example, by applying the formulas of Eqs. (5) and (6) for Element 2 (with uniform load *q*=-*w*), we get:

9 $$x_{{extrM}} = - b \cdot \frac{{2a - 2L + b}}{{2L}}$$

10 $$M(x_{{extrM}} ) = \frac{{b^{2} w(2a - 2L + b)^{2} }}{{8L^{2} }} - \frac{{abw(2a - 2L + b)}}{{2L}}$$

**Fig. 9:**
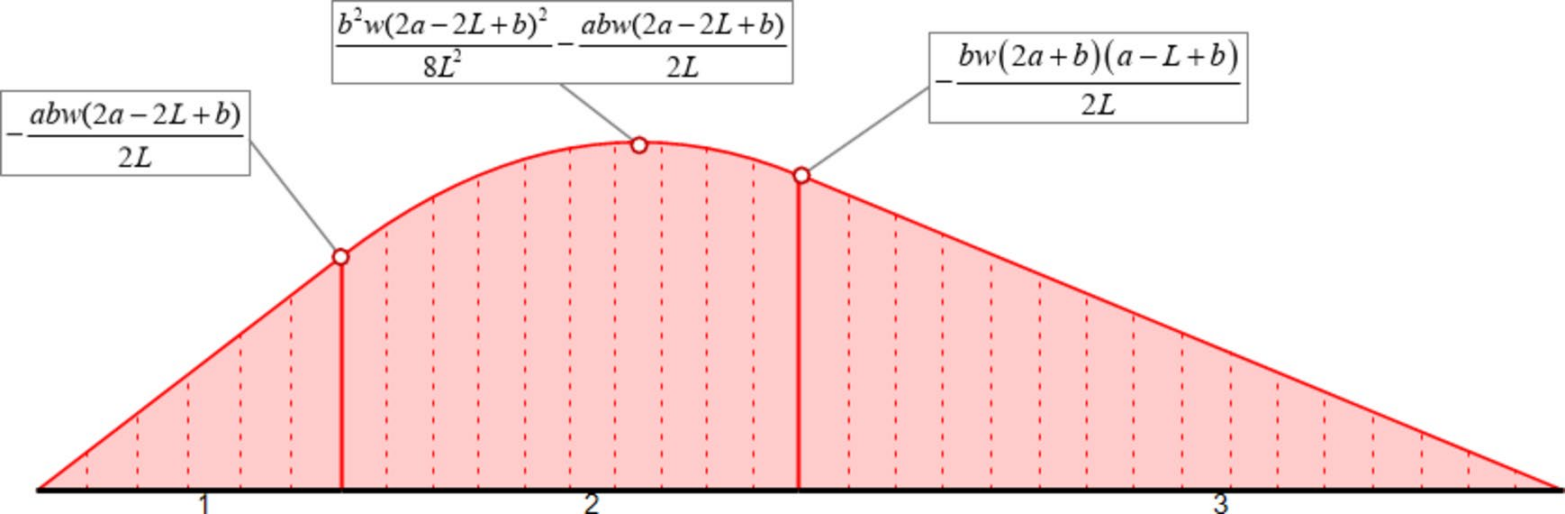
Symbolic bending moment diagram for Example 4.

We see that we get the exact value of the maximum bending moment along Element 2, in a symbolic form. By combining the results presented in Table 18 and Eqs. (9) and (10), we can draw the bending moment diagram of the beam in a symbolic way, including the maximum bending moment at the span 2, as shown in Fig. 9.

### Four-Span beam with uniform load (3 symbolic Parameters)

**Fig. 10:**
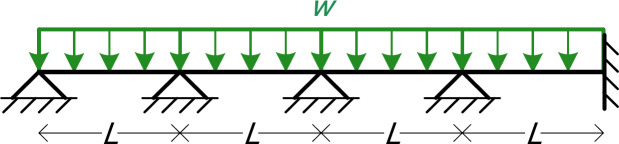
The four-span beam of Example 5.

The fifth numerical example is a four-span continuous beam with a uniform load *w*, fixed at its right end, where all spans have the same length *L*, as shown in Fig. 10. Although the computational model is larger and more complex than the previous ones and there are more nodes and elements, the symbolic parameters in this example are only three: *EI*, *L*, and *w*.

Table 19 shows the details of the model, as given in MATLAB. In this example we have 5 nodes and 4 elements.

**Table 19:** Details of the input parameters of the 5th numerical example.

Lengths	$${\left[ {\begin{array}{*{20}{c}} L&L&L&L \end{array}} \right]^T}$$
Supports	$${\left[ {\begin{array}{*{20}{c}} 1&1&1&1&1 \\ 0&0&0&0&1 \end{array}} \right]^T}$$
PointLoads	$${\left[ {\begin{array}{*{20}{c}} 0&0&0&0&0 \\ 0&0&0&0&0 \end{array}} \right]^T}$$
UniformLoads	$${\left[ {\begin{array}{*{20}{c}} { - w}&{ - w}&{ - w}&{ - w} \end{array}} \right]^T}$$

The results of the symbolic analysis are given in Tables 20 and 21, and Table 22, for Node displacements and rotations; support reactions; and element forces and moments, respectively.

**Table 20:** Example 5: node displacements and rotations.

Node #	y-Displacement (D_y_)	z-Rotation (*R*_z_)
Node 1	0	$- \frac{{7w{L^3}}}{{291EI}}$
Node 2	0	$\frac{{5w{L^3}}}{{776EI}}$
Node 3	0	$- \frac{{w{L^3}}}{{582EI}}$
Node 4	0	$\frac{{w{L^3}}}{{2328EI}}$
Node 5	0	0

**Table 21:** Example 5: support reactions.

Node #	Force F_y_	Moment M_z_
Node 1	$\frac{{153wL}}{{388}}$	-
Node 2	$\frac{{110wL}}{{97}}$	-
Node 3	$\frac{{187wL}}{{194}}$	-
Node 4	$\frac{{98wL}}{{97}}$	-
Node 5	$\frac{{193wL}}{{388}}$	$- \frac{{8w{L^2}}}{{97}}$

**Table 22:** Example 5: element forces and bending moments.

Element #	Start / End	Shear Force (V_i_, V_j_)	Moment (M_i_, M_j_)
1	Start (i)	$\frac{{153wL}}{{388}}$	0
	End (j)	$\frac{{235wL}}{{388}}$	$- \frac{{41w{L^2}}}{{388}} \approx - 0.105670w{L^2}$
2	Start (i)	$\frac{{205wL}}{{388}}$	$\frac{{41w{L^2}}}{{388}} \approx - 0.105670w{L^2}$
	End (j)	$\frac{{183wL}}{{388}}$	$- \frac{{15w{L^2}}}{{194}} \approx - 0.077320w{L^2}$
3	Start (i)	$\frac{{191wL}}{{388}}$	$\frac{{15w{L^2}}}{{194}} \approx - 0.077320w{L^2}$
	End (j)	$\frac{{197wL}}{{388}}$	$- \frac{{33w{L^2}}}{{388}} \approx - 0.085052w{L^2}$
4	Start (i)	$\frac{{195wL}}{{388}}$	$\frac{{33w{L^2}}}{{388}} \approx - 0.085052w{L^2}$
	End (j)	$\frac{{193wL}}{{388}}$	$- \frac{{8w{L^2}}}{{97}} \approx - 0.082474w{L^2}$

In this example, by applying the formulas of Eqs. (5) and (6) for all elements (as all elements have a uniform load *q*=-*w* applied on them), we get the results presented in Table 23 for the maximum value of the internal bending moment for each span.

**Table 23:** Location and value of max. Bending moment for each span of the beam (5th numerical example).

Element #	x_extrM_	M(x_extrM_)
1	$\frac{{153L}}{{388}}$	$\frac{{23409w{L^2}}}{{301088}} \approx 0.077748w{L^2}$ *
2	$\frac{{205L}}{{388}}$	$\frac{{{\text{10209}}w{L^2}}}{{301088}} \approx 0.033907w{L^2}$
3	$\frac{{191L}}{{388}}$	$\frac{{{\text{13201}}w{L^2}}}{{301088}} \approx 0.043844w{L^2}$
4	$\frac{{195L}}{{388}}$	$\frac{{{\text{12417}}w{L^2}}}{{301088}} \approx 0.041240w{L^2}$

*Max value at any span.

**Fig. 11:**
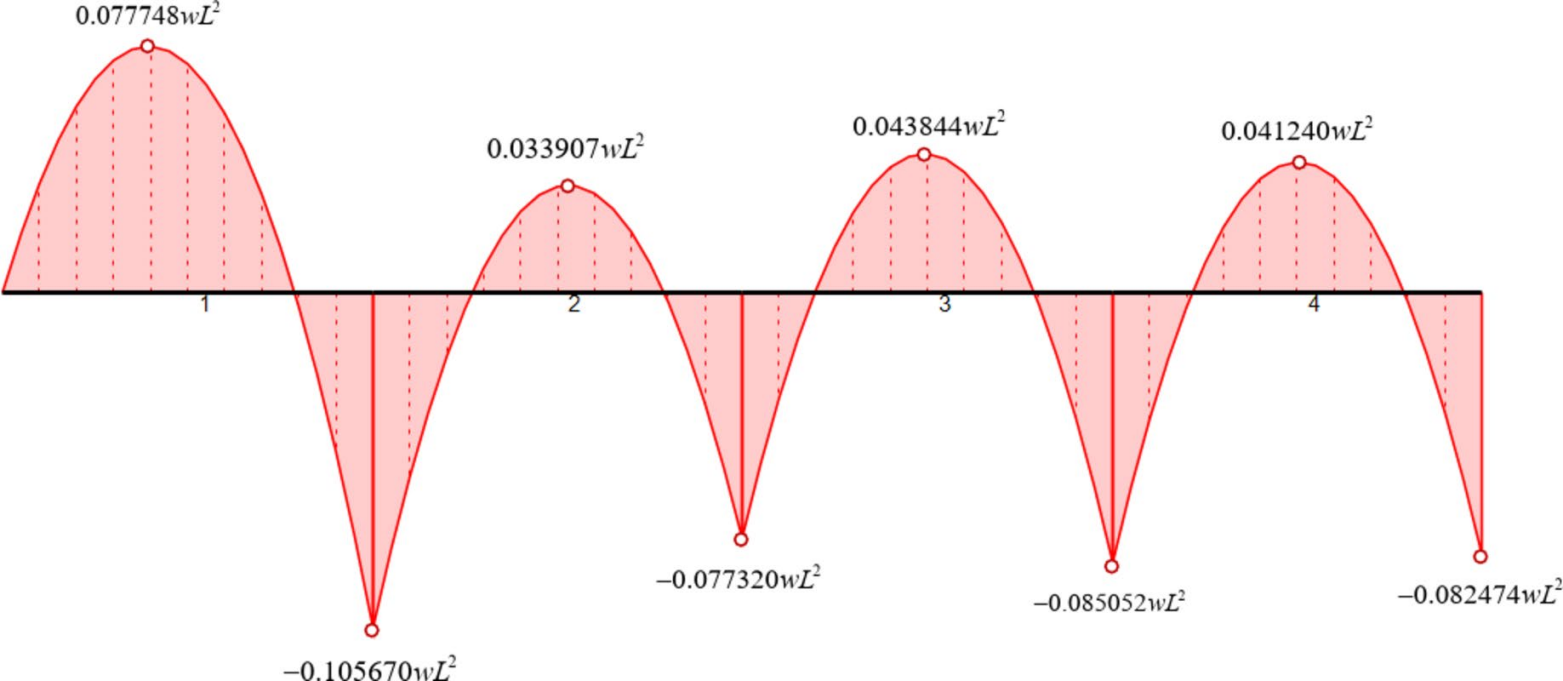
Symbolic bending moment diagram for Example 5.

By taking the results presented in Table 22 (for element end values) and Table 23 (for span values), we can draw the bending moment diagram of the beam in a symbolic way, as shown in Fig. 11.

## Sensitivity analysis

Sensitivity analysis is a powerful technique used in structural engineering to evaluate how variations in input parameters affect the system’s response. It plays an important role in understanding the robustness of a design and in optimizing performance by identifying which parameters have the most significant impact on the system’s behavior. One benefit of the application of symbolic solutions to problems in structural engineering is that sensitivity analysis becomes straightforward. If the behavior of a structural system is expressed symbolically, the engineer has access to closed-form expressions that explain exactly how changes in such important parameters such as beam length, modulus of elasticity, or loads will influence the system response.

This makes it easy to compute partial derivatives of any output with respect to any input parameter. For example, with a symbolic expression for displacement, one can easily compute the sensitivity of the displacement to changes in beam length. For example, consider the second numerical example shown in the previous section. The vertical displacement at *x* = *a*, where the load *P* is applied, according to Table 8 is given by

11 $$D_{{y,x}} = - \frac{{Px^{2} (L - x)^{3} (3L + x)}}{{12EIL^{3} }}$$

Using mathematics or the MATLAB Symbolic Math Toolbox and its commands *diff* and *simplify*, we can compute the partial derivative of the displacement with respect to any parameter (*P*, *x*, *L*, *EI*), giving us a mathematical expression for how changes of a parameter affect the displacement. In this example, the partial derivative of *D*_y, x_ with respect to *L* can be found as:

12 $$\frac{{\partial {D_{y,x}}}}{{\partial L}}= - \frac{{P{x^2}{{({L^2} - {x^2})}^2}}}{{4EI{L^4}}}$$

This partial derivative provides the sensitivity of the displacement to changes in beam length, offering insight into how modifications in the design will affect the performance of the structure. The same approach can be applied to assess the sensitivity of internal forces, bending moments, or support reactions to variations in other input parameters.

In numerical analysis, results are obtained for specific input values, requiring repeated analyses to assess parameter sensitivity. In contrast, symbolic expressions retain input-output relationships, enabling direct sensitivity evaluation without additional computation. This provides engineers with deeper insight, allowing them to predict how design changes will affect structural behavior. This capability is particularly valuable in optimization, where small adjustments can significantly impact performance and cost, eliminating the need for repeated numerical simulations and enhancing design efficiency.

## Efficiency, scalability and elegance of symbolic computations

Although the symbolic implementation of MatSA can offer significant advantages, it also presents challenges in terms of computational efficiency and scalability, particularly for larger systems. As structural systems grow in complexity, the symbolic representations of stiffness matrices, force vectors, and displacement fields can become increasingly large and computationally demanding. It is important to address these challenges to ensure that symbolic MatSA remains practical and efficient for engineering applications.

For a simple system, a symbolic equation may provide a clear and elegant solution. However, for larger systems or those involving a large number of symbolic variables, the resulting expressions can quickly become excessively long and complex. A symbolic solution that spans multiple pages with dense expressions will offer little practical value compared to a simple numerical result. This is a critical limitation because, even though symbolic software like MATLAB will always attempt to generate a symbolic solution, the elegance and simplicity of the solution are important for engineers.

To deal with this problem, a practical approach is to limit the number of symbolic variables by keeping only some key parameters while handling less critical ones numerically. This hybrid symbolic-numerical approach keeps expressions compact and comprehensible, balancing flexibility with computational efficiency. A well-structured symbolic solution should be concise, interpretable, and insightful; if overly complex, it loses practical value despite being technically correct. By keeping the right balance, engineers can use symbolic computation’s advantages without excessive complexity, ensuring both clarity and efficiency in analysis.

Table 24 presents the computation times for the five examples, with the second column showing the total time required to run each example 100 times and the third column displaying the average time per run, calculated by dividing the total time by 100. All computations were performed on a Windows 11 Pro (24H2) desktop with 64 GB RAM, a 4 TB NVMe SSD, and a 12th Gen Intel^®^ Core™ i7-12700 F processor (2.10 GHz). The results illustrate the increasing computational cost as beam complexity grows. Although symbolic computations provide valuable analytical insights, users should be mindful of this trade-off when applying the method to larger beam systems.

**Table 24:** Computational time needed to run each of the examples.

Example #	Time for 100 runs (s)	Average time for a single run (s)
1	5.93	0.06
2	10.50	0.11
3	21.48	0.21
4	22.42	0.22
5	12.74	0.13

## Conclusions

This study has presented the development and application of an open-source MATLAB program capable of performing symbolic matrix structural analysis for continuous beams under point and uniform loads. The tool, freely available on GitHub, enables users to generate analytical solutions efficiently and accurately for various beam configurations. These solutions are valuable in both engineering practice and education, offering insights into structural behavior while complementing numerical methods.

Beyond deriving analytical solutions for displacements, support reactions, and internal forces, the program can also generate influence lines for continuous beams and facilitate sensitivity analysis, which is particularly beneficial in optimization and design exploration. The ability to compute partial derivatives of output parameters with respect to input variables provides an enhanced understanding of how design choices influence structural responses.

Although symbolic solutions offer clarity and parameterized insights, they should always be viewed as a complementary approach rather than a replacement for numerical methods. Numerical methods remain essential for large-scale structural problems involving thousands or millions of unknowns, where symbolic computation is impractical. Symbolic MatSA, on the other hand, provides explicit mathematical relationships, making it particularly useful for exploring parameter dependencies, conducting theoretical studies, and reinforcing fundamental structural mechanics concepts.

Additionally, one must recognize the trade-off between symbolic precision and computational complexity. The program is capable of producing detailed symbolic expressions, but in some cases, overly complex formulas may reduce practical applicability. Balancing elegance, clarity, and efficiency is key when utilizing symbolic computations in engineering applications.

Through various examples, we have demonstrated the potential of symbolic MatSA in providing insightful analytical solutions. The next step in this research is to extend the methodology to other structural systems, such as 2D and 3D trusses and frames, further expanding the scope of symbolic structural analysis. By integrating symbolic and numerical methods, engineers and researchers can achieve a more comprehensive understanding of structural behavior, benefiting both computational analysis and engineering education.

## Data Availability

The source code of the program is freely available in the GitHub repository (https://github.com/vplevris/SymbolicMatSA-Beams), accompanied by all the examples discussed in the study.
